# Delta-range coupling between prefrontal cortex and hippocampus supported by respiratory rhythmic input from the olfactory bulb in freely behaving rats

**DOI:** 10.1038/s41598-021-87562-8

**Published:** 2021-04-14

**Authors:** Rola Mofleh, Bernat Kocsis

**Affiliations:** Department Psychiatry at BIDMC, Harvard Medical School, 3 Blackfan Circle, Boston, MA 02215 USA

**Keywords:** Cognitive neuroscience, Neural circuits, Neurological disorders, Neuroscience, Neurology

## Abstract

Respiratory rhythm (RR) during sniffing is known to couple with hippocampal theta rhythm. However, outside of the short sniffing bouts, a more stable ~ 2 Hz RR was recently shown to rhythmically modulate non-olfactory cognitive processes, as well. The underlying RR coupling with wide-spread forebrain activity was confirmed using advanced techniques, creating solid premise for investigating how higher networks use this mechanism in their communication. Here we show essential differences in the way prefrontal cortex (PFC) and hippocampus (HC) process the RR signal from the olfactory bulb (OB) that may support dynamic, flexible PFC-HC coupling utilizing this input. We used inter-regional coherences and their correlations in rats, breathing at low rate (~ 2 Hz), outside of the short sniffing bouts. We found strong and stable OB-PFC coherence in wake states, contrasting OB-HC coherence which was low but highly variable. Importantly, this variability was essential for establishing PFC-HC synchrony at RR, whereas variations of RRO in OB and PFC had no significant effect. The findings help to understand the mechanism of rhythmic modulation of non-olfactory cognitive processes by the on-going regular respiration, reported in rodents as well as humans. These mechanisms may be impaired when nasal breathing is limited or in OB-pathology, including malfunctions of the olfactory epithelium due to infections, such as in Covid-19.

## Introduction

More than half a century after the first observations^1^, an explosion of findings firmly demonstrated that brain activity and cognitive function in rodents and humans are modulated synchronously with nasal respiration (rev.^2,3^). Respiratory related oscillations (RRO) were detected in numerous brain structures, including higher order cognitive centers in the prefrontal cortex (PFC)^4,5^ and hippocampus (HC)^6–8^. RRO coupling with wide-spread forebrain activity was recently confirmed using advanced techniques, including current source density^6,8,9^, single unit firing^4–6,9,10^, and phase modulation of local gamma activity^4,5,11–13^. Respiratory rhythm primarily derives from airflow through the nasal cavity providing rhythmic input to the olfactory bulb (OB)^7^, which dynamically couples with intrinsic network oscillations in these structures either: (1) by coherence, when the frequency of RRO matches that of local field potentials such as delta and theta activity in rodents^4,6–8,11,14^, or (2) by phase-amplitude modulation when the frequencies diverge, as in gamma oscillations in rodents^4,5,7,11^. In human, where respiratory rate (< 0.5 Hz) is out of the frequency range of most rhythms in the electroencephalogram (EEG), relevant for cognitive function (delta, theta, alpha and gamma oscillations), RRO is also established using the mechanism of phase-amplitude modulation^15^.

Accumulating evidence over the past decade has advanced research on the mechanisms underlying OB-cortical RRO coupling from well-studied sniffing episodes, to mechanisms associated with continuous on-going respiration – thereby raising questions about how RRO may be involved in non-olfactory cognitive processing^3,16^. Respiratory modulation of a wide range of cognitive functions has been reported both in rodents and human, from sensory processing and motor coordination to various memory functions (rev.^3^) – which are not directly related to olfaction or to gas exchange (as a primary respiratory function). Rhythmic coupling is a powerful, ubiquitous mechanism of functional coordination of neural ensembles, and RRO appears to be a potential source of wide, perhaps even global^2,14^ synchronization of various networks, cortical as well as subcortical. For functional networks, access to rhythmic drive has to be dynamically regulated in a state- and task-dependent manner to encompass specific circuits involved in particular tasks – to couple them when necessary and uncouple them when it is not. The anatomical systems carrying the RRO signal to diverse networks appear suitable to exert such control. RRO from different sources^17^, of which OB is dominant, is transmitted to various networks which may differentially synchronize with this input dependent on their unique circuit characteristics and connectivity. For example, key intrinsic oscillations in PFC (delta, 2–5 Hz^18–20^) are in the range of on-going RRO, whereas those in the HC are faster (theta, 5–10 Hz^21^), overlapping in rodents with sniffing frequency. These two forebrain structures are the focus of the current study investigating in the rat how the RRO signal generated in the OB may potentially contribute to PFC-HC communication by synchronizing their activities at the respiratory rate.

Effective PFC-HC communication is important for normal cognition and impaired PFC-HC coupling was implicated in cognitive deficits in psychiatric diseases^22–24^. We have shown recently in rats that rhythmic coupling between PFC and HC can be established in both delta and theta ranges, even simultaneously, and proposed that they may serve as parallel channels of communication (but in opposite directions) between the two structures^25^. RRO and theta was shown to co-exist during theta states with an asymmetric regional distribution in the cortex; that is, RRO dominant in the frontal cortex and theta in more caudal cortical areas^14^. Based on these data we hypothesized that RRO might enhance the communication between PFC and HC networks primarily in the PFC-to-HC direction. Indeed, we found a strong and reliable RRO transmission through the OB to PFC whereas OB-HC coherence was low in all states. RRO in HC was highly variable but showed strong correlation with RRO coherence between PFC and HC. Thus, PFC delta output was steadily segmented and shaped by the RRO, but it was the HC response to the common RRO drive that dynamically regulated PFC-HC coupling in the delta range. The details of this regulation remain unknown. Importantly however, the strong effect of RRO on cortical coupling suggests that damage to OB may lead to functional abnormalities in higher brain function. It is known for example that SARS-CoV-2 viral infection of ACE2 receptor-expressing epithelial cells related to the OB leads to loss of smell^26^, associated with significant alteration in brain imaging and correlated with not only smell but also with memory loss^27^. A similar mechanism may affect the OB-dependent RRO as well.

## Results

### Respiratory rhythm in diaphragmic electromyogram (dia EMG) correlates with LFP oscillations in OB

**Figure 1:**
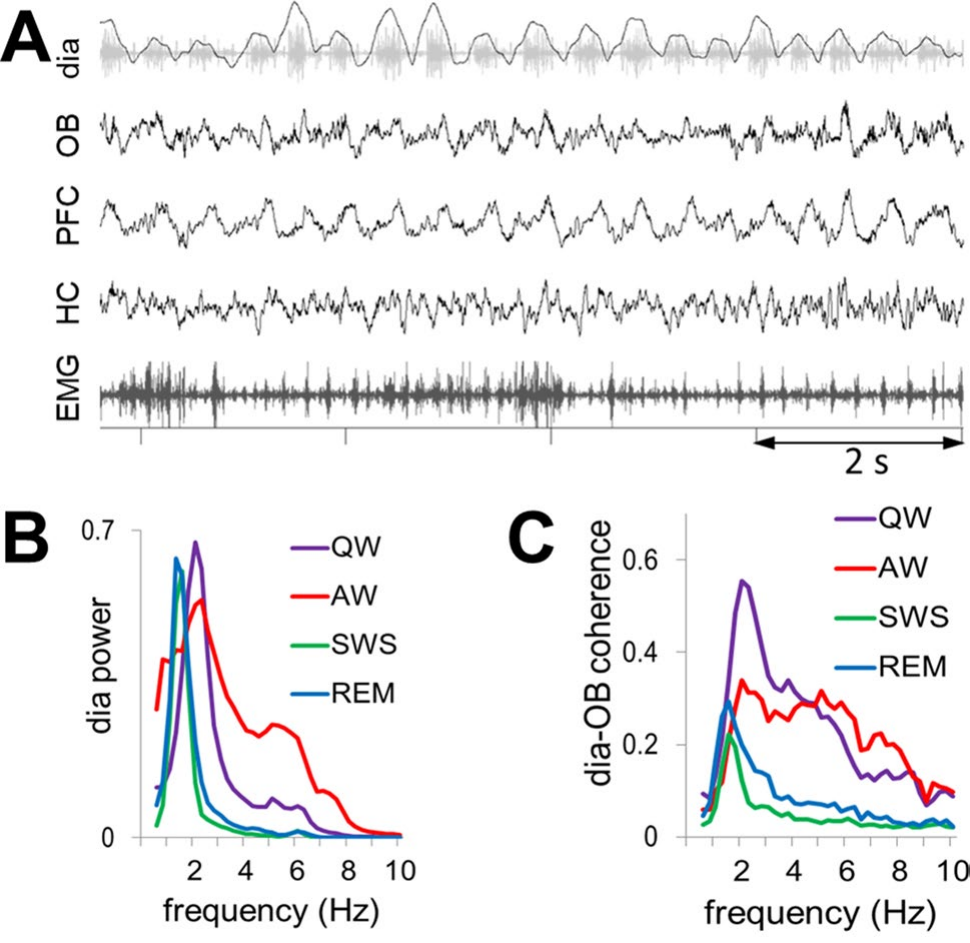
(**A)** Sample recording of respiratory rhythm (black, inspiration up) derived from diaphragmal EMG (gray) along with LFPs in OB, PFC, and HC and neck muscle EMG in QW state. (**B)** Group averages of dia autospectra in different states. Note narrow RRO peaks in all recordings at ~ 2 Hz. Power is shown in arbitrary units after normalization of autospectra in individual recordings setting maxima equal to 1. (**C)** Group averages of dia-OB coherence spectra in different states. Note coherence peaks constrained to RRO frequencies (i.e. dia spectral peaks) in sleep and in a wider range, up to 6 Hz in wake states. In AW, dia-OB coherence does not have a clear RRO peak on the group average due to interindividual variability of the respiratory rates (see in Fig. S2).

Respiratory rate varies extensively in rats, covering the entire range of characteristic frequencies of low frequency oscillations (delta, theta, even alpha) intrinsically generated by neural circuits in the cortex and hippocampus^2^. To focus on on-going regular RROs (Fig. 1A), the present analysis was limited to lasting stationary segments – excluding short segments with fast RROs potentially associated with sniffing (Fig. S1). Thus, respiratory rate (RRO frequency) was in the delta range in all states – below 2 Hz in sleep, and slightly faster in waking, but still below the theta range (Table S1). Except for active waking (AW), RRO was stable in all states, manifested by a single sharp peak in the autospectra of the dia EMG signal in each recording session (Fig. S2A). RRO frequency in these states (quiet waking – QW, rapid eye movement sleep – REMs, slow wave sleep – SWS), shifted from experiment to experiment in a narrow range, producing ~ 1 Hz-wide peaks in average dia autospectra (Fig. 1B). LFP in the OB was correlated with this signal in a state-dependent manner (see below) giving rise to RRO peaks in the dia-OB coherence spectra, which in sleep were also restricted to this narrow frequency range (Fig. 1C).

In waking, RRO peaks in dia autospectra were somewhat wider in each experiment (indicating short-time scale variations within recording sessions) and its peak shifted in a wider range between experiments (1–3 Hz; Fig. 1B). In a few recordings (4 in AW and 1 in QW), there was a second dia power peak at 4–6 Hz (Fig. S2A), but this always appeared together with a clearly distinguishable 1–3 Hz component (Fig. S1C). The lower peak (1–3 Hz) was also present in the group averages of dia power spectra (Fig. 1B) and dia-OB coherence functions (Figs. 1C and S2B).

To examine the origin of RRO coupling between higher order networks, we used pairwise coherences between dia, OB, PFC, and HC signals and their correlations, calculated at the respiratory frequency, in all states. Respiratory rate was identified from dia autospectra, in each individual experiment.

### OB unevenly conveys RRO to higher order networks in PFC and HC

**Figure 2:**
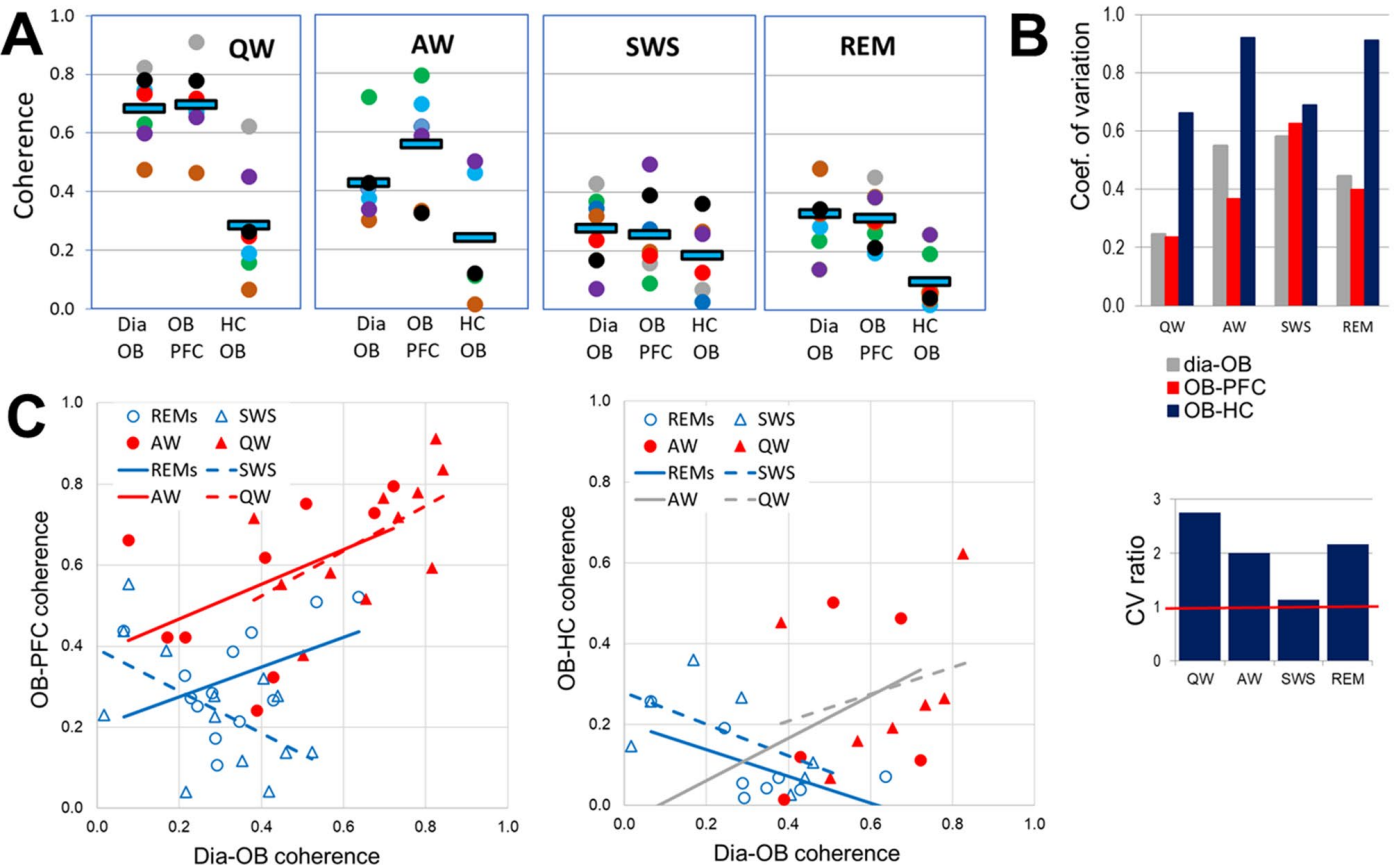
Comparison of state-dependent RRO coherences in PFC and HC transferred through OB. (**A)** Pair-wise coherences at the respiratory frequency (RRO) between rhythmic dia activity and LFP in the OB and between OB and cortical (PFC) and hippocampal (HC) networks during different sleep–wake states. Note strong state dependence and nearly identical dia-OB and OB-PFC coherences in all recordings and considerably lower OB-HC coherence. Squares: group averages, dots: individual experiments; same colors identify individual rats across different states. (**B)** Variability of coherence values in individual experiments in different states. Coefficient of variation (top) and CV ratio (bottom) of coherences in the OB-HC vs. the other two signal pairs (dia-OB and OB-PFC). Note high variation of OB-HC in waking (AW and QW) and REM sleep, 2–3 times exceeding CV of the other pairs. (**C)** Relationship between RRO coherences connecting dia to OB C(Dia-OB) and those connecting OB to neural networks of PFC (left) and HC (right) in different states. Trend lines with nonsignificant correlations (p > 0.1) are shown in gray; solid lines show theta, and dashed lines show non-theta states. Note the significant positive correlation between dia-OB and OB-PFC but no positive correlation between dia-OB and OB-HC coherences (only a non-significant trend in waking).

To assess RRO synchronization across regions, RRO coherences between signal pairs representing RRO transfer from rhythmic nasal airflow to the OB and then from OB to PFC and HC, were compared in different behavioral states. dia-OB coherences showed strong state dependence (Figs. 1C and 2A, Table 1A) and while RRO coherence between OB and PFC followed this pattern, those between OB and HC were relatively low in all states (Fig. 2A). Thus, OB-PFC coherences at the frequency of respiration (RRO) were higher during wake than sleep states; differences between AW and QW vs. SWS and REMs were all statistically significant (p < 0.01), whereas within waking and within sleep no significant differences were detected (p > 0.1). On the other hand, group averages of OB-HC coherence were in a narrow range; they did not change between QW, AW, and SWS and were somewhat lower in REM sleep (only significant at the level of p < 0.1). In all states, OB-PFC coherences were similar to dia-OB coherence (i.e. statistically equal, p > 0.1), in major contrast to the pathway conveying RRO to HC; where OB-HC and dia-OB coherences were significantly different in all states (p < 0.01), except SWS.

**Table 1:** Relationship between coherences through OB and their effect on RRO synchronization between PFC and HC. Italic highlights relatively high coherences, above 0.4, and significant correlations (p < 0.05), R^2^, between pairwise coherences. C(X–Y): coherence between X and Y.

RRO Coherence	SWS	REM	QW	AW
**A. Coherences connecting OB to dia and LFP signals of PFC and HC at RRO frequency**				
C(Dia-OB)	0.29 ± 0.04	0.33 ± 0.04	*0.66* ± *0.05*	*0.40* ± *0.07*
C(OB-PFC)	0.25 ± 0.04	0.32 ± 0.04	*0.67* ± *0.05*	*0.55* ± *0.07*
C(OB-HC)	0.18 ± 0.05	0.09 ± 0.03	0.29 ± 0.07	0.24 ± 0.10

R^2^ between C(PFC-HC) and…	SWS	REM	QW	AW
**B. Correlations between C(HC-PFC) and coherences connecting OB to other signals**				
C(Dia-OB)	0.08	0.01	0.00	0.00
C(OB-PFC)	0.04	0.07	0.33	0.12
C(OB-HC)	0.27	0.00	*0.69*	*0.79*

Peak coherence	SWS	REM	QW	AW
**C. Coherences connecting LFP signals of PFC and HC at RRO frequency**				
C(PFC-HC)	0.22 ± 0.04	0.19 ± 0.07	*0.45* ± *0.08*	*0.42* ± *0.09*

Examination of pairwise coherences in individual experiments further supported the pattern revealed by group averages. OB-HC coherence was lower than dia-OB and OB-PFC in all experiments in all states (see colors of dots, representing different experiments in Fig. 2A), even though RRO coherences showed natural variation between experiments. Furthermore, the variability of coherence values revealed a feature, unique for the OB-HC relationship, further separating it from dia-OB and OB-PFC. When comparing different states, a drop in averages was normally associated with decrease in standard deviation, as well, for both OB-PFC and OB-HC (p < 0.04 in F-tests comparing variances in QW or AW with SWS or REM). In contrast, OB-HC variances were similar to other signal-pairs (F-test, p > 0.05), despite significant differences in averages coherences. Thus, the coefficient of variation (CV, a measure of relative variability; Fig. 2B) reflected widely dispersed values of OB-HC coherences, compared with the other signal-pairs in QW and AW. CV of OB-HC exceeded that of dia-OB and OB-PFC by 100–200% (CV ratio > 2; Fig. 2B).

To study the origin of this variability, dia-OB coherences quantifying RRO input to the OB were correlated with RRO coherences in the pathways connecting OB further to the PFC and to the HC. Specifically, a strong correlation would indicate that the more RRO is derived by OB from rhythmic nasal airflow, the more it is transferred further, to its downstream targets. For the PFC, this was indeed the case; we found that dia-OB and OB-PFC coherences were positively correlated (Fig. 2C) in all sleep–wake states (R > 0, p < 0.1; Table S2), except SWS where the correlation was negative. In contrast, no such faithful, obligatory transmission of RRO through OB was found for the HC. No significant correlation between dia-OB and OB-HC coherences was detected in waking (see non-significant trendlines marked grey in Fig. 2C), and the correlation was negative in sleep states. Thus, the consistent increase in RRO conveyed by the OB to the PFC was closely associated with RRO variation in the OB network derived from respiration (Fig. 2C), whereas RRO transmission from OB to HC, varying in a wide range from one experiment to the next (Fig. 2B), did not follow the variations of dia-OB coherence in waking (AW and QW) and it was in fact showing an opposite tendency in sleep states (Fig. 2C).

### Role of OB-mediated RRO in coupling between higher order networks in PFC and HC

**Figure 3:**
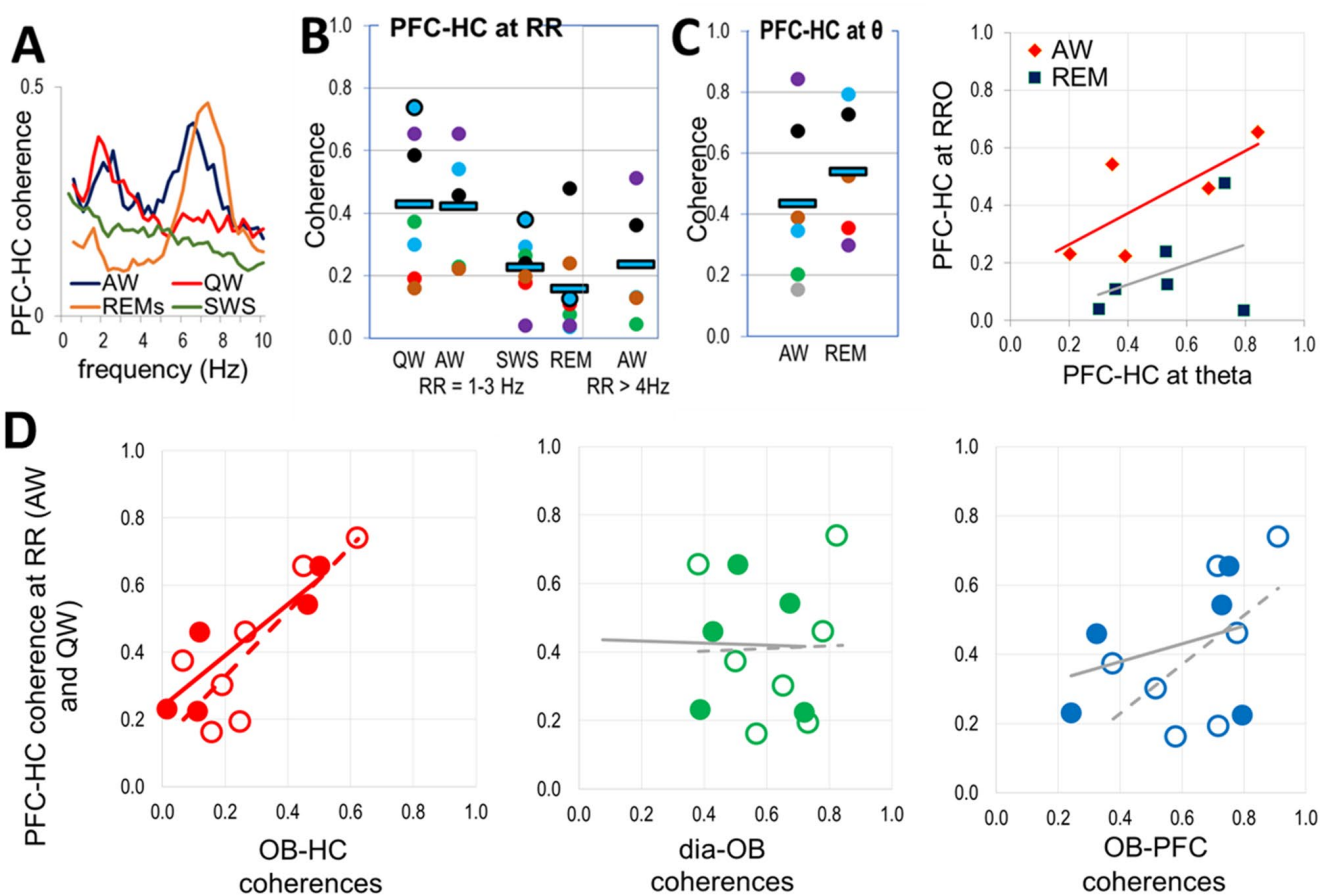
Coupling of PFC and HC networks by RRO. (**A)** PFC-HC coherences at the respiratory frequency (RRO) in different states. (**B)** PFC-HC coherences at RRO in awake (QW, AW) and sleep states (SWS, REM) at RRO (1–3 Hz). Squares: group averages, dots: individual experiments; same colors were used for individual rats in different states (same colors as in Fig. 2A). (**C)** Distribution of PFC-HC coherences at theta (6–8 Hz) frequency and it’s relationship to RRO coherences in theta states. (**D)** Correlation between RRO coherences connecting PFC and HC vs. RRO coherences connecting OB to HC, dia, and PFC signals in AW (filled symbols and solid trendlines) and QW (open symbols and dashed trendlines) recordings at RRO frequency. Significant correlations are shown in the color of the corresponding dots, and trendlines of nonsignificant correlations are shown in gray.

Oscillatory coupling between PFC and HC was reported in different states in the rat, both in delta and theta frequency bands^20,24,25^, as maintained by various mono- and polysynaptic connections between the two structures. Theta peaks were also dominant in PFC-HC coherence spectra during theta states (AW, REM sleep) in the present study. By adding simultaneous dia EMG and OB recordings, however, we could also identify coherences indicative of PFC-HC coupling at respiratory frequency in the delta range. RRO coherence peaks appeared either alone (QW) or in addition to theta (AW; Fig. 3A).

Average RRO coherence between PFC and HC fell in between OB-PFC and OB-HC coherence values in all states (Table 1C), although the differences were only significant in QW (p = 0.02) – with a sufficient gap between OB-PFC and OB-HC coherences. PFC-HC coherence was significantly higher in waking (above 0.42) than sleep (below 0.22) and did not change significantly within these states (i.e. p > 0.1 for AW vs. QW and REM vs. SWS comparisons) (Fig. 3B). The relationship of OB-HC < PFC-HC < OB-PFC coherences was robust; it was also valid in most individual experiments (60 and 71% of recordings in AW and QW, respectively). When both present, PFC-HC coherences at theta and RRO frequencies showed parallel variations between experiments (Fig. 3C; R^2^ = 0.54 and 0.43, p = 0.04 and 0.07 in AW and REM, respectively).

Unlike theta rhythm, primarily generated in HC and conveyed to PFC^28^, producing strong PFC-HC coherence, the origin of the RRO coherence between these structures is less certain. RRO is generated outside of these structures, and from the OB it is faithfully transmitted to the PFC but much less reliably to the HC (Figs. 2A, 2C).

RRO coherence in the PFC-HC signal-pair may be due to RRO received by PFC from OB and then transmitted to HC or may be the effect of common input from OB to PFC and HC. The former appears consistent with relatively strong, state-dependent RRO in OB-PFC and PFC-HC coherences (compare Figs. 2A and 3B), and the latter with the large variability of RRO transmission to HC (Fig. 2B), i.e. relatively high in some experiments and lower in others in wake states (Fig. 2A).

To distinguish between these possible mechanisms, we next compared correlations between RRO coherences in individual experiments in each state in which RRO coherences were present (Fig. 3D). We investigated in particular, whether stronger RRO synchrony between HC and PFC signals was associated with stronger OB-PFC or with stronger OB-HC coherences. As shown in Table 1B and Fig. 3D, PFC-HC RRO coherence significantly correlated only with RRO coherences linking OB with HC but not with PFC. This relationship was found in all states showing strong RRO. Thus, R^2^ correlation coefficient revealed similarity between the variations of PFC-HC and OB-HC coherences from one experiment to the next in QW (R^2^ = 0.69, p = 0.003) and in AW (R^2^ = 0.79, p = 0.01; Table 1B).

In contrast, PFC-HC coherence did not correlate with OB-PFC coherence in any state (Table 1B). A positive trend was observed in QW (R^2^ = 0.33) but was not significant (p = 0.06; see grey lines in Fig. 3D). Variations of PFC-HC coherence in individual experiments was not affected by variations of dia-OB coherence in waking states and by any coherence connecting dia, OB, PFC, HC signals during sleep (Figs. 3D, S4).

## Discussion

This study used inter-regional coherences and their correlations to trace the RRO signal in freely behaving rats from OB, where it is derived from rhythmic nasal airflow, to higher order brain networks of PFC and HC, where it may potentially contribute to communication between these structures by synchronizing their activities at the respiratory rate. We focused on on-going (i.e. “background”) RRO unaffected by behaviors requiring its short-term fluctuations, e.g. sniffing. We found that this rhythmicity depends on sleep–wake states; it is significantly larger in waking than in sleep (see also^29^). Within arousal states, however, it remains unchanged when the animal is engaged in behaviors or conditions, such as locomotion (AW), and REM sleep associated with theta vs. consummatory behaviors (QW) and SWS associated with non-theta HC activity. In agreement with previous reports^4,5,14,30^, RRO was more prominent in PFC with an obligatory transmission of RRO from OB to PFC. This was indicated by parallel variations in dia-OB and OB-PFC coherences in individual experiments, verified by significant correlation between these parameters over the group. In contrast, RRO was relatively low in HC, and the variations of OB-HC coherence did not necessarily follow those between dia and OB. RRO input to HC^6–8^, however, lead to strong variations in individual recordings (at odds with the grossly defined states of AW and QW). Importantly, this variability, quantified with OB-HC coherence, was essential for establishing PFC-HC synchrony at the respiratory rate, whereas variations of RRO in OB and PFC had no significant effect.

### RRO in waking

Communication and collaboration between HC and PFC and its impairment in psychiatric diseases has been the primary focus of extensive research to date^22,23,28,31^. HC-PFC theta synchronization and its role in spatial working memory is relatively well-studied, in rats, mice and in humans^32–37^. On the other hand, PFC is theorized to be the master regulator of working memory and higher-order executive function^38–43^, yet the mechanisms by which PFC exerts “top-down” influences remain less clear. Functional coupling of PFC with downstream circuits, including HC, amygdala, ventral tegmental area, by means of delta-range oscillations (2–5 Hz) was recently shown in PFC-specific tasks^18–20^. It was proposed that theta and delta oscillations may serve as parallel channels of communication between the HC and the PFC in opposite directions: theta HC-to-PFC and delta PFC-to-HC^25^. The results of the present study indicate that the balance and interaction between theta and RRO may also provide a potential mechanism for bidirectional PFC-HC coupling. When RRO is within the delta range, it can contribute to or even drive the PFC 2–5 Hz rhythm^44,45^. PFC receives this input whenever OB is driven by RRO and may broadcast it widely. In contrast, the connection of HC to this global^2^ rhythm appears more dynamic; when the HC is receptive to RRO input, PFC-to-HC channels would open in the delta range – distinct from the channel established by HC-theta in the opposite direction.

The most striking observation of this study was the marked contrast between the strength and reliability of RRO input, stronger in PFC than in HC (Table 1A), and its effect on PFC-HC coherence, i.e. RRO in HC more influential than in PFC (Table 1B). This raises questions regarding the mechanisms and the functional consequences. Differences in cytoarchitecture, connectivity, and other properties of PFC and HC networks give rise to different intrinsic oscillations in the two structures, which allow them to resonate with rhythmic input at specific frequencies. We propose that, due to these differences, baseline slow RRO recorded in this study may be involved in PFC-HC communication primarily in the of PFC-to-HC direction.

Task-related intrinsic oscillations in PFC were shown in rats and mice at frequencies in the delta range^18–20^, but these oscillations in waking are markedly different from the wide-band thalamo-cortical delta rhythm of SWS. Specifically, the delta (of waking) is spectrally of narrow-band, is hierarchically nested with gamma oscillations^24,46^, and is normally generated in cortico-cortical circuits, associated with various cognitive functions^46–49^. Outgoing PFC messages may use the RRO fluctuations in sensitivity of downstream structures when PFC delta and RRO are synchronized. With this mechanism, RRO outside of sniffing^6–8^ may make HC networks sensitive to messages arriving assembled in bouts at delta-range frequencies.

On the other hand, background RRO is slower than HC theta rhythm and thus, in order to synchronize with the signature HC oscillation during active states of sniffing, respiratory rate is accelerated and brought within the higher and narrower theta frequency band. The two oscillations, RRO and theta, show distinct characteristics in HC, such as different laminar profiles and theta-modulated gamma bands, and differentially entrain HC neurons^6–8^ – even when their frequencies overlap. Olfactory-related activity patterns in OB, such as cell firing and gamma bursts, appear phase locked in these episodes to the synchronized RRO-theta rhythm^50^. The exact mechanisms are not completely understood, but rhythmic synchronization of sensory sampling in OB on one hand and excitability of neurons involved in central processing in HC and piriform cortex on the other is considered a “paradigmatic example” of active sensing^16,51^. This would serve to optimize odor perception, coordinating it with multiple sensory channels, associated with rhythmic nasal, whisker, and head movements.

Although lacking strong direct projections from the OB^7,52,53^, the PFC and HC receive RRO via multisynaptic pathways which includes common connections from the piriform cortex, as well as separate projections, through amygdala (PFC) or entorhinal cortex (HC)^4,7^. Accordingly, the PFC-HC coherence may emerge from common OB input, or alternatively the RRO may be directed primarily to PFC and then transmitted to HC. Differential nodes in OB output pathways connecting PFC and HC (see e.g.^25,54^) may set the balance of RRO between the two structures.

When and how HC couples with slow baseline RRO will require further investigations using specific tasks beyond the sleep–wake states of this study (see e.g.^29^). Data demonstrating the potential role of RRO in non-olfactory processing has accumulated in recent years, not only from rodent studies but also from human studies^15,55,56^. A specific challenge for translating the results between species resides with important differences between human and rodents. Brain oscillations, functions, dynamics, and key features including characteristic frequencies are evolutionarily well-preserved^57^, but respiratory rate varies widely between species. In humans, respiratory rate (~ 0.2 Hz) is below the frequencies of the key components of the EEG oscillatory hierarchy which thus cannot establish coherent coupling with respiration. RRO remains however manifested in humans as respiratory modulation of the amplitude of brain oscillations including slow (delta, theta) as well as fast (beta, gamma) rhythms – involved in cognitive processes^15^. This is a different form of coupling which unlike coherence does not require matching the frequencies of rhythms generated by different mechanisms.

### RRO in sleep

In contrast to wake states, RRO during sleep appears reduced at the level of OB indicated by relatively low dia-OB coherence (Fig. 2) thus restricting OB-mediated RRO in higher brain structures (PFC, HC) from coupling with oscillations dominant in these networks during sleep. Viczko et al.^58^ demonstrated for example in the rat that slow oscillations (SO), an archetypical EEG pattern in SWS, emerges separate from respiration even when they overlap in frequency, and argued that it “fits with an SO mechanism as intrinsic emergent property of a deafferented neural network”. Our data are consistent with this concept, suggesting that intrinsic brain oscillations – relevant in sleep-dependent memory consolidation both in SWS (SO^58^ and delta^59^) and REM sleep (theta^60^) – are protected from RRO. It is interesting to note that in humans, where very few studies analyzed RRO during sleep with statistical scrutiny^61,62^, subtle changes in EEG linked to respiratory cycles were enhanced in SWS and REM sleep in children with sleep disordered breathing in multiple frequency bands, including delta^61^, theta^61,62^, alpha, and sigma^62^. Adeno-tonsillectomy, the most common surgical procedure for sleep apnea, which among other benefits improves cognitive function, reduced or normalized these RRO alterations.

It is important to note, however, that our conclusions only concern rhythmic RRO mediated by the OB. It has been reported that, besides RRO, respiration may also pace non-rhythmic events, linking their occurrence to specific phases of respiration (see e.g.^17,55^). In sleep, this may include sharp wave/ripples and dentate spikes; that is, intrinsic HC patterns during SWS synchronized with UP-DOWN transitions in cortical networks that are involved in functional PFC-HC interactions serving memory consolidation^63,64^. For instance, a recent study^65^ found in mice that the post-inspiratory bias of these patterns along with firing of a large number of PFC and HC neurons remained after deafferentation of OB sensory neurons, indicating that mechanisms that bypass the OB play a primary role in their synchronization. The authors hypothesized that the contribution of a “so-far undescribed ascending respiratory corollary discharge signal, likely propagating from the brainstem respiratory rhythm generators” could pace limbic networks using a disinhibition-mediated mechanism – consistent with lack of prominent LFPs in the absence of input from the OB^65^. The causal model remains to be elucidated. In addition to ascending projections from the pre-Bötzinger complex^66^ or the locus coeruleus^67^, several other signals from internal organs may be involved –due to respiratory movements and chemosensitive signals from the cardiovascular system^17,68,69^.

There may be further patterns of cortical activity modulated by respiration during sleep which would not be revealed by analysis of LFP coherence. REM sleep for example, is characterized by strong theta-gamma coupling^70,71^ which was recently reported to vary according to changes in instantaneous respiratory rate^72^. Theta rhythm and RRO are independent of one another; in the HC they generate dipoles in different layers^6,8^. Their frequencies differ in REM sleep and thus the two oscillations do not phase-lock^5,6^. They might however correlate in REM sleep through mechanisms other than phase-entrainment effects.

### Potential relevance to COVID-19

We are not aware of published research on whether impaired RRO mechanisms are implicated in COVID-19 pathology. However, disturbances in smell, emerged early as a predominant neurological symptom^73,74^, serve as evidence for COVID-19 related neurological abnormalities originating from pathology of the olfactory epithelium. According to current understanding (rev.^26,75^), SARS-CoV-2 does not directly infect olfactory sensory neurons; their deficit is mediated instead by the altered microenvironment maintained by cells in the olfactory epithelium expressing ACE2 receptors^76–78^. Yet, the potentially lasting damage is not localized to the OB, in the first prospective imaging studies (magnetic resonance imaging-MRI scans 3–4 months after COVID-19 hospitalization), significant changes in grey matter volume were primarily found in cingulate gyrus, piriform cortex, and hippocampus, correlated with loss of smell and also with memory loss^27^.

Much less is known about the cellular mechanism of RRO generation in the OB but conditions similar to those leading to smell loss may also affect RRO. Olfactory sensory neurons can respond not only to odorants but also to mechanical stimuli^79,80^ and transmit both odor and air flow-driven mechanical signals^81,82^. The latter was only studied however in mechanisms and schemes related to sniffing while their role in low frequency RRO, targeting a wide range of forebrain regions, remains unidentified. Involvement of non-sensory cells of the olfactory epithelium in RRO remains not clear either, but their impaired function in providing structural support, maintenance of ionic environments, etc. may negatively affect RRO, as well.

To date, we have solid data demonstrating that RRO depends on OB mechanisms^2,7,11^ and modulates higher brain function^15,55,56^. Abnormal PFC-HC coupling would not be immediately noticeable for patients, as the more obvious symptom of smell loss is, but may lead to neurological consequences. Further studies are necessary to address the effect of SARS-CoV-2 on this system.

## Methods

Male rats (360–560 g, Charles River Laboratories) were used in this study. Experiments were performed on 8 rats subjected to survival surgery followed by chronic recordings in free behaviors. All procedures were approved by the Institutional Animal Care and Use Committee of Beth Israel Deaconess Medical Center and carried out in accordance with relevant guidelines and regulations. The study and reporting also adheres to ARRIVE guidelines.

### Experimental procedures

Diaphragmal EMG was recorded in all rats along with LFP in the OB, PFC and HC using microwires (Fig. S5) and EEG using screw electrodes over the parietal cortex with additional EMG recordings in the neck muscle, all referenced to a screw electrode placed over the cerebellum (see Supplementary Information for details). Recordings were made in undisturbed condition. Recordings started 7–10 days after surgery in two 24 h recording sessions, acquired a couple of days apart in each rat. Sleep–wake states (AW, QW, REMs, and SWS) were identified using standard criteria based on cortical EEG, HC LFP, and neck muscle EMG recordings. For analysis, multiple segments were selected from discontinuous episodes of each state dispersed over the 2 days of recordings, in which respiration appeared relatively stable without fluctuations (Table S1). Respiratory rate varied in different states in a relatively narrow band (between 1–3 Hz, Table S1) with an occasional faster component (4–6 Hz) in AW which did not overlap with theta frequency, specifically verified in each segment submitted for analysis.

### Data analysis

Was performed on recordings acquired at 1 kHz. Dia EMG recordings were processed using built-in procedures of Spike2 to remove electrocardiogram (ECG) contamination and to convert high-frequency EMG components in order to retrieve pure respiratory rhythm. Noise-free segments with stable respiration for at least ~ 100 s were selected in SWS, REM, QW and AW, recorded on two different days (see Table S1 for the number and length of segments in each state), and submitted to Fast Fourier Transform to obtain power spectra and coherence function with ~ 0.25 Hz frequency resolution. As a measure of the correlation between signals in the frequency domain, we used coherence; prior studies referenced above also used coherence^6–8,11–14,19,20,22,23,30^ or phase locking which excludes amplitude correlation^4,10,35^. Coherence values were compared against chance using surrogate-based statistical testing^14^ and potential contamination due to volume conduction or common reference was tested using cross-correlations in the time domain (see more details in Supplement, Fig.S6). To quantify neuronal synchronization between different structures we used pairwise coherences, calculated between 4 signal pairs, representing the potential transfer of the RRO signal to higher-order structures through the OB (i.e. dia with OB and OB with PFC and HC) and between these higher order structures (i.e. PFC with HC). Power spectra for dia EMG and HC were also calculated to identify the frequencies of spectral peaks of respiration (RRO) and theta rhythm. Coherence values at RRO and theta frequencies were calculated in each segment (Table S1) and daily averages of these values were used in statistical analysis, including group averages (Tables 1 and S2) and comparisons of RRO coherences. Differences between coherences in different states were tested using Student’s t-test after Fisher r to z transformation to obtain z-scored values with normal distribution, thus allowing parametric statistical techniques. Correlation between pair-wise coherences was statistically tested using Excel’s T-DIST procedure (see more details in Supplement).

## Supplementary Information

Supplementary Information

## Abbreviations

RRO: Respiratory related oscillations
LFP: Local field potentials
EEG: Electroencephalogram

## Anatomical terms

PFC: Prefrontal cortex
HC: Hippocampus
OB: Olfactory bulb
Dia: Diaphragm

## Sleep–wake states

AW: Active waking
QW: Quiet waking
SWS: Slow wave sleep
REMs: REM (rapid eye movement) sleep
