# Supplementary material for: Delta-range coupling between prefrontal cortex and hippocampus supported by respiratory rhythmic input from the olfactory bulb in freely behaving rats
Source: Sci Rep. 2021 Apr 14;11:8100. doi: 10.1038/s41598-021-87562-8 (PMC8046996; doi:10.1038/s41598-021-87562-8)
Supplement: Supplementary file 1 — Supplementary Information [file 41598_2021_87562_MOESM1_ESM.pdf]

Supporting Information for

Delta-range coupling between prefrontal cortex and hippocampus supported by respiratory rhythmic input from the olfactory bulb in freely behaving rats

Rola Mofleh and Bernat Kocsis

Dept Psychiatry at BIDMC, Harvard Medical School
Correspondence: bkocsis@hms.harvard.edu

This PDF file includes:

Figures S1 to S6

Tables S1 and S2

Detailed Experimental Procedures

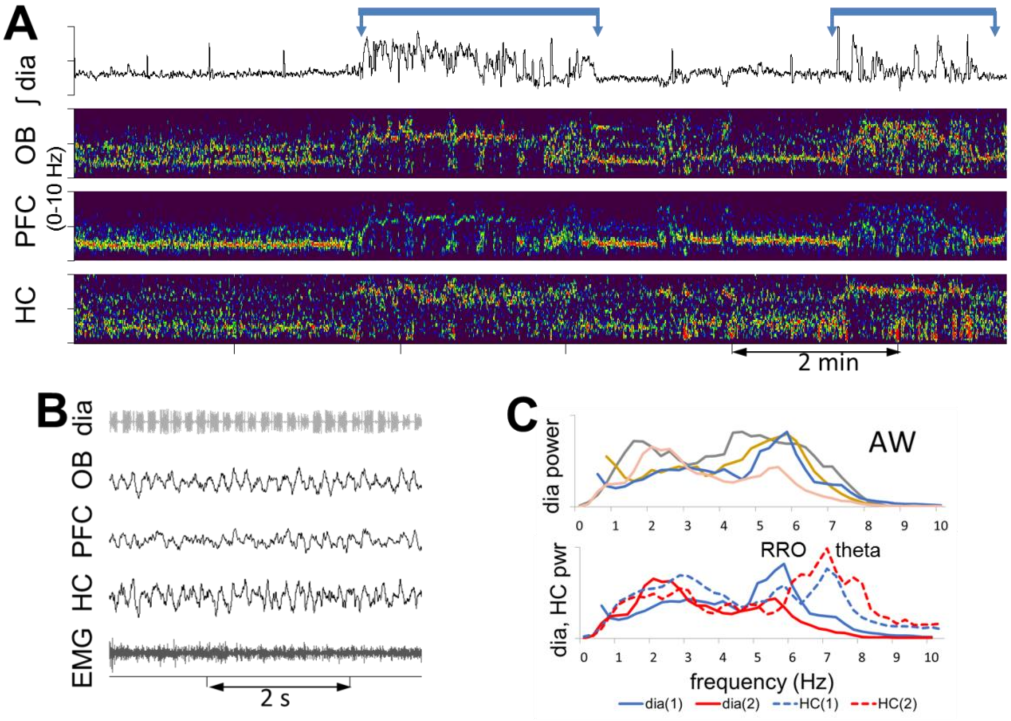

Supplementary Figure S1. Unstable RRO in AW. Example of segments with RRO matching theta frequency, presumably associated with sniffing and excluded from analysis in this study (see marked in A) and segments with slow and faster RRO not synchronized with theta frequency (B). **A**. Rapidly alternating episodes of slow and fast respiration, presumably associated with sniffing. Respiration (integrated dia EMG) and time-frequency plots of LFP signals are shown in the 0-10 Hz range. Note matching frequencies of different signals in slow RRO and theta segments. **B**. Example of theta segment with RRO at theta frequency. **C.** *Top:* Two spectral peaks in dia autospectra, one at 2-3 Hz, the other at 4-6 Hz, in 4 out of 10 AW recordings. *Bottom:* Dia (solid lines) and HC (dashed lines) autospectra showing that high RRO did not overlap with HC theta.

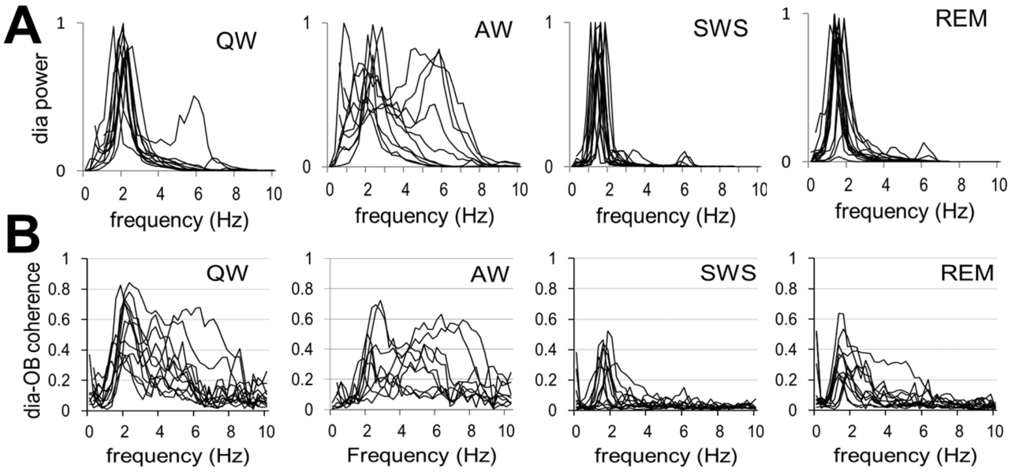

Supplementary Figure S2. RRO oscillations in dia EMG, correlated with OB LFP. **A.** Autospectra of dia EMG in individual experiments. Note single sharp RRO peaks varying in a narrow frequency range in all states, except AW which showed larger variations. **B**. dia-OB coherence spectra in individual experiments. Note dia power peaks at higher frequencies in a few recordings (1 in QW and 4 in AW) and more frequently in dia-OB coherences, which appeared in addition to an obligatory slow component below 4 Hz,.

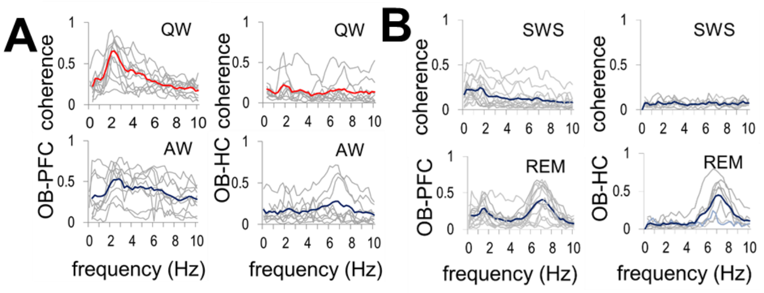

Supplementary Figure S3. Coherence functions (0-10 Hz) between OB and higher order networks of PFC and HC during waking (**A**; QW and AW) and sleep (**B**; SWS and REM) in individual experiments (grey) and averaged over the group (red and blue). Note high RRO coherence in waking in the range of respiratory frequency and low RRO coherence sleep, along with strong theta peak during REM sleep.

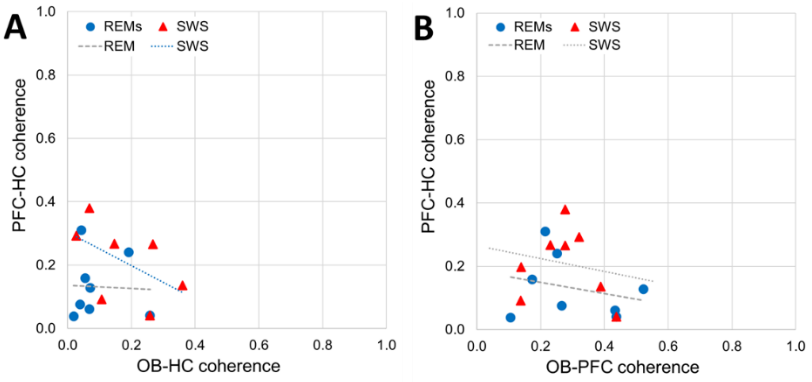

Supplementary Figure S4. Correlation between coherences at the respiratory rate (RRO) between PFC and HC, and RRO coherences connecting OB to HC (**A**), and PFC signals (**B**) in different states with weak or non-existent RRO (REM sleep, SWS). Significant correlations are shown in the color of the corresponding dots, trendlines of non-significant correlations are shown in grey.

Supplementary Table S1: length of segments and average RRO and theta frequency in different states

		SWS	REM	QW	AW
in each rat (2 days)	Ave # of segments	9 ± 0.8	9 ± 0.9	7 ± 0.4	7 ± 0.5
	Ave length (s)	127 ± 11	109 ± 12	112 ± 18	95 ± 14
	Total length (s)	1237 ± 219	1031 ± 186	821 ± 147	670 ± 98
# rats(recordings) with identified RRO in LFP		7(14)	7(14)	7(12)	6(9)
RRO frequency (Hz)		1.65 ± 0.05	1.65 ± 0.05	2.01 ± 0.08	2.23 ± 0.11
and range (Hz)		1.2—2.0	1.2—2.0	1.5—2.4	1.0—2.9
Theta freq. (Hz)			7.08 ± 0.09		6.84 ± 0.13
and range (Hz)			6.8—7.3		6.6—7.1

Supplementary Table S2: Correlation between dia-OB coherences and OB-PFC and OB-HC (p<0.1 positive correlations are marked with yellow background,). C(X-Y): coherence between X and Y.

R2 between C(dia-OB) and…	SWS	REM	QW	AW
… C(OB-PFC)	0.31 (-)	0.17	0.33	0.21
… C(OB-HC)	0.35 (-)	0.41 (-)	0.08	0.12

Experiment procedures

Male rats (360–560g, Charles River Laboratories) were used in this study. Experiments were performed on 8 rats subjected to survival surgery followed by chronic recordings in free behaviors. All procedures were approved by the Institutional Animal Care and Use Committee of Beth Israel Deaconess Medical Center and carried out in accordance with relevant guidelines and regulations. The study and reporting also adheres to ARRIVE guidelines.

Survival surgeries were conducted in sterile conditions under deep anesthesia, maintained by a mixture of Ketamine-Xylazine (i/p; 30-40 mg/kg ketamine and 5 mg/kg Xylazine) with supplementary injections of Ketamine (10 % of the initial dose) if necessary. Stainless steel screws above the parietal cortex (AP: -3.5 mm, Lat: 2.5 mm from bregma) were inserted on the left side to record the cortical EEG and in the nasal bone (~5.0 mm anterior to bregma) and above the cerebellum to act as ground and reference electrodes. Two single electrodes (stainless steel wires) were implanted on each side (AP: +3.2 mm, Lat: ±0.5 mm, DV: -5.1 mm) to record the electrical activity in the PFC, a single electrode on the left side for the OB (AP: +8.5 mm, Lat: 1.5 mm, DV: -1.6 mm), and a pair of twisted wires with 1 mm between their tips in the HC on the right side (AB: -3.7, Lat: 2.2 mm, DV: -3.5 mm) (Fig. S5). All the electrode wires and screws were fixed to the skull with dental acrylic. In addition, multi-threaded electrodes in soft insulation were implanted in the diaphragm to record diaphragmal EMG and in neck muscles to evaluate general motor activity and tone to identify sleep-wake states, and channeled under the skin to the head-connector. The electrodes were connected to an amplifier (A-M systems) for data recording, filtered between 0.3 and 100 Hz, whereas the dia EMG filters had both low- and high-pass filters set at 100 Hz to diminish unwanted noise including respiratory movement artifacts and electrical noise from the heart. Antibiotic gel was applied before suturing the incisions. Meloxicam analgesics, 1 mg/kg of 5 mg/ml daily, was given subcutaneously two days in a row to relieve pain. The rats were observed until they fully woke up. They recovered for a week in their home cages before the first 24 hours-recording. These recordings included different behaviors and sleep-wake states while the rat was freely moving in the cage, undisturbed. The rats were euthanized by 1 ml of Ketamine injections 3 weeks later and the brains were extracted for verification of the electrode position (Fig. S5).

Data and statistical analysis

In two 24 hr recordings, acquired a couple of days apart in each rat, sleep-wake states were identified using standard criteria based on cortical EEG, HC LFP, and neck muscle EMG recordings. In waking, characterized by high and variable muscle activity, active waking (AW) and quiet waking (QW) were separated by the presence or absence, respectively, of HC theta rhythm and low amplitude fast cortical EEG. Sleep periods, characterized by continuously low muscle tone were also divided by the presence of HC theta accompanied by low-amplitude cortical EEG (REM sleep) or large amplitude slow activity dominating cortical and HC recordings (slow wave sleep, SWS). For analysis, multiple segments were selected from discontinuous episodes of each state dispersed over the 2 days of recordings, in which respiration appeared relatively stable without fluctuations (Table S1). Respiratory rate varied in different states in a relatively narrow band (between 1-3 Hz, Table S1) with an occasional faster component (4-6 Hz) in AW which did not overlap with theta frequency, specifically verified in each segment submitted for analysis.

EEG (filtered between 0.3 and 100 Hz) and EMG (100-300 Hz) recordings were acquired as a ~.DDF-files in DASYLab 7.0 (at 1 kHz sampling rate) and then imported to Spike2 (Version 7.06, Cambridge Electronic Devices) for signal analysis. Dia EMG recordings were processed using built-in procedures of Spike2 to remove ECG contamination and to convert high-frequency EMG components to retrieve pure respiratory rhythm.

Noise-free segments with stable respiration for at least ~100 s were selected in SWS, REM, QW and AW, recorded on two different days (see Table S1 for the number and length of segments in each state), and submitted to Fast Fourier Transform (FFT) to obtain power spectra and coherence functions. FFT was performed on consecutive 4.096 s windows, using a program (COHER.S2S) from the Spike2 library. FFT converts a block of waveform data into frequency representation; the FFT of a block of n data points sampled at a frequency F produces n/2+1 equally spaced complex numbers ($A_f = a\ e^{j\varphi}$), from a frequency of 0 to F/2, spaced by F/n. Power spectral density of each signal and cross spectral density for signal pairs are calculated using these complex

numbers, as $psd(f) = a\ e^{j^{\varphi}}\ a\ e^{-j^{\varphi}} = a^2$ and $csd(f) = a\ e^{j^{\varphi}(a)}\ b\ e^{-j^{\varphi}(b)} = a\ b\ e^{j(^{\varphi}a-^{\varphi}b)}$. To quantify neuronal synchronization we used coherence analysis calculated using these psd and csd-s, as $coh(f) = (SUM(csd_{ab}(f))^2 / SUM(psd_a(f)) * SUM(psd_b(f))$, where SUM(.) denotes summation over blocks of data.

Coherence function is calculated for pairs of different signals of interest (e.g., LFP recorded from two spatially distinct areas) and provides a useful measure of synchrony between two signals at each frequency of the spectrum, thus revealing characteristic frequencies of synchronization. Since coherence is essentially computed using cross spectral density estimate, this connectivity metric depends on both the magnitude and phase of the signals at hand. Pairwise coherences were calculated between 4 signal pairs, representing the potential transfer of the RRO signal to higher-order structures through the OB (i.e. dia with OB and OB with PFC and HC) and between these higher-order structures (i.e. PFC with HC). Power spectra for dia EMG and HC were also calculated to identify the frequencies of spectral peaks of respiration (RRO) and theta rhythm in individual experiments. Coherence values were compared against chance using surrogate-based statistical testing by computing coherence spectra between PFC and HC vs. OB of the same rat, as well as OB from a different animal recorded at the same time and processed in parallel using different channels of the same amplifier, A/D converter, and stored in the same file. The significance of coherence values depends on the length of the analyzed segment (see different levels of erroneous coherence in examples of short and long segments in Fig. S6A-B) but was statistically different (lower) in all signal pairs ($p<0.0001$ for OB-PFC and PFC-HC in all states, as well as for OB-HC in QW and SWS; $p=0.025$ and $p=0.001$ for OB-HC in REM and AW, respectively). To verify that the RRO coherences were not driven by the common reference over the cerebellum or by volume conduction, cross-correlation was calculated on a QW segment which showed high RRO coherence, in each rat. The phases between different LFPs were inconsistent between experiments, but the RRO peaks did not overlap (see examples in Fig. S6C-E). Differences between coherences in different states were tested using Student's t-test after Fisher r to z transformation to obtain z-scored values with normal distribution. Fisher transformation allows using parametric techniques to test hypotheses about the value of the population correlation coefficient and coherences as the sampling distribution of the resulting variable is approximately normal, with a variance that is stable over different values of the underlying true correlation. Correlation between pair-wise coherences at the respiratory rate were statistically tested using Excel's T-DIST procedure using the formula p=TDIST(R*SQRT((N-2)/(1-R2)),N,1) where R is coherence and N is number of experiments, included.

Perfusion and Histology.

Perfusion was conducted by inserting a hypodermic needle connected to a pump into the ascending aorta through the left ventricle of the heart. The right ventricle was cut open and phosphate buffered saline (PBS) was pumped into the circulation system for 5 minutes, until all blood had been drained and replaced with PBS. Then 10% buffered formalin solution was pumped for about 15-20 minutes, until the rat was stiff. After perfusion, the rats' brain was carefully extracted and placed in a vial filled with 20 mL of formalin for further fixation for at least one hour, while stored at 4ºC. Before cryosectioning (Microm, HM 450), the brains were stored in 20% sucrose solution overnight at 4 °C until sank to the bottom of the vial. 45 µm slices were stored in PBS with Azide and then mounted on slides and stained with Cresyl Violet, coverslipped and evaluated to locate the marks of damage due to inserted recording electrodes (Fig. S5).

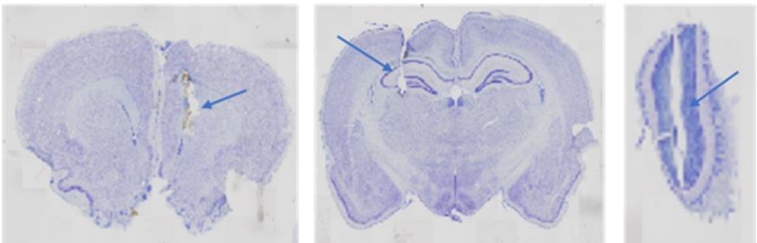

Supplementary Figure S5. Placement of electrodes for local field potential (LFP) recordings. Histology of stained coronal sections to identify electrode coordinates in the prefrontal cortex (PFC), hippocampus (HC), and olfactory bulb (OB).

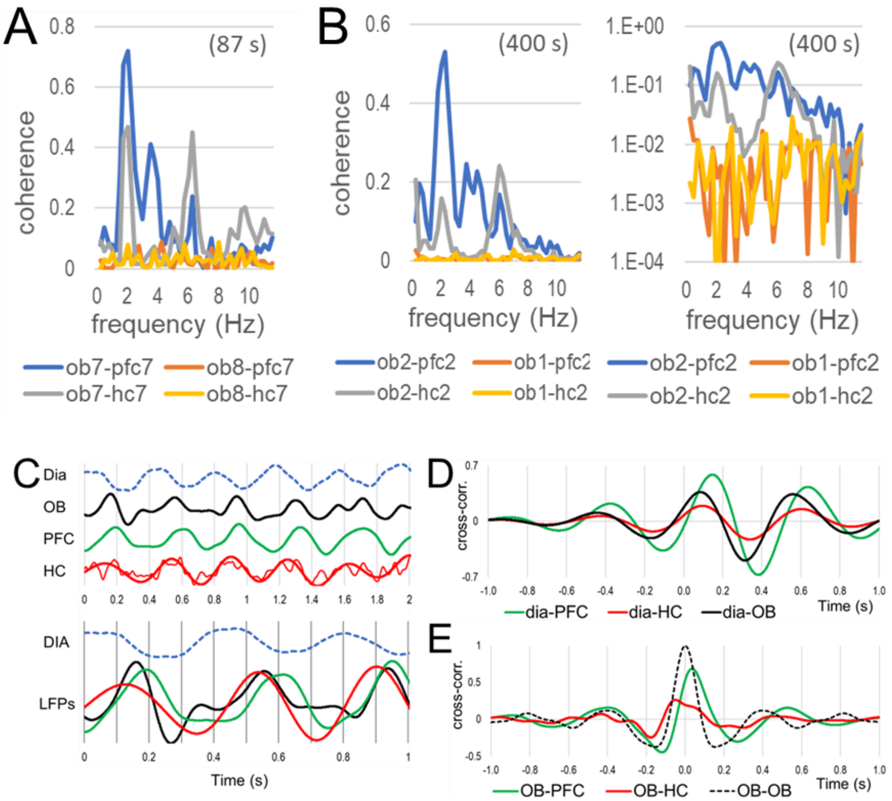

Supplementary Figure S6. Coherence analysis. **A-B.** Comparison of OB-PFC (*blue*) and OB-HC (*grey*) coherences with coherences values calculated using a surrogate signal (OB of a different rat, recorded simultaneously, *red* and *yellow*) in a short (A) and long segment (B, shown in linear and log scale). **C-E.** Phase relationship between RRO in different LFPs. **C**. *Top*: Short (2 s) sample recording of dia, OB, PFC, and HC in QW. LFPs were filtered below 18 Hz (HC was additionally filtered below 3 Hz to obtain a visually smooth trace for illustration). *Bottom*: same traces (LFPs overlapped) on different time scale. **D**. Sample cross-correlograms between dia EMG and PFC, HC, and OB LFPs in QW. **E**. OB autocorrelogram (dashed line) and cross-correlogram between OB and higher order (PFC and HC) LFPs. B and C are from the same QW segment.
